# Iron metabolism in breast cancer: mechanisms and therapeutic implications: a narrative review

**DOI:** 10.1097/MS9.0000000000003173

**Published:** 2025-03-20

**Authors:** Emmanuel Ifeanyi Obeagu, Mahamane Salissou Maibouge Tanko

**Affiliations:** Department of Biomedical and Laboratory Science, Africa University, Mutare, Zimbabwe

**Keywords:** breast cancer, ferritin, iron chelation, iron metabolism, therapeutic targeting

## Abstract

Iron metabolism plays a crucial role in breast cancer progression by supporting rapid cell proliferation, metastasis, and therapy resistance. Breast cancer cells exhibit increased iron uptake through overexpression of transferrin receptor 1, leading to intracellular iron accumulation. Elevated ferritin levels further protect tumor cells from oxidative stress-induced damage, while reduced ferroportin expression limits iron export, creating an iron-rich microenvironment that favors tumorigenesis. These alterations in iron homeostasis contribute to an aggressive cancer phenotype and are associated with poor clinical outcomes. Beyond its role in tumor growth, dysregulated iron metabolism influences the tumor microenvironment by promoting angiogenesis and immune evasion. Iron-laden macrophages within the tumor stroma support cancer progression by supplying iron to malignant cells, fueling their metabolic demands. Moreover, iron-driven oxidative stress generates reactive oxygen species, leading to DNA damage, genomic instability, and the activation of pathways involved in epithelial-mesenchymal transition. These processes enhance breast cancer cell invasion and metastasis, particularly in aggressive subtypes such as triple-negative breast cancer.

## Introduction

Breast cancer remains the most commonly diagnosed malignancy among women worldwide, with its incidence steadily rising due to genetic, environmental, and lifestyle factors. While significant advancements have been made in understanding the molecular mechanisms driving breast cancer, metabolic alterations, including iron dysregulation, have emerged as critical contributors to tumor initiation, progression, and therapy resistance. Iron, an essential trace element, plays a fundamental role in cellular proliferation, energy metabolism, and DNA synthesis. However, its dysregulation in breast cancer creates a pro-tumorigenic environment that fuels malignant transformation and disease progression^[1–6]^. Iron homeostasis is tightly controlled under normal physiological conditions to balance iron uptake, storage, and export. However, in breast cancer, this regulation is often disrupted, leading to excessive iron accumulation within tumor cells^[7]^. Breast cancer cells exploit iron metabolism by overexpressing transferrin receptor 1 (TfR1), the key mediator of iron uptake, thereby enhancing intracellular iron levels to meet their high metabolic demands^[7]^. Elevated TfR1 expression has been correlated with increased tumor aggressiveness, rapid proliferation, and poor prognosis, particularly in estrogen receptor-negative and triple-negative breast cancer (TNBC) subtypes^[8]^. In addition to increased iron uptake, alterations in iron storage mechanisms further contribute to breast cancer progression. Ferritin, the major intracellular iron storage protein, is frequently upregulated in breast cancer cells, allowing them to sequester excess iron and protect against oxidative stress-induced damage. This adaptation provides a survival advantage by mitigating iron-dependent cytotoxicity while maintaining a readily available iron reservoir to support continuous tumor growth. Notably, higher ferritin levels in breast cancer patients have been associated with increased resistance to chemotherapy and radiation therapy^[8]^.

**HIGHLIGHTS**

- Altered iron homeostasis: Breast cancer cells exhibit dysregulated iron metabolism, contributing to tumor growth, and metastasis.
- Iron’s role in angiogenesis: Iron influences angiogenesis, supporting tumor vascularization, and survival in hypoxic environments.
- Therapeutic targeting: Targeting iron metabolism pathways offers potential for novel treatments, including iron chelators, and inhibitors.
- Iron and tumor microenvironment: Iron influences immune cells and oxidative stress within the tumor microenvironment.
- Resistance mechanisms: Breast cancer cells can develop resistance to therapies targeting iron metabolism.

Iron export is another critical aspect of iron metabolism that is frequently dysregulated in breast cancer^[9]^. The iron-export protein ferroportin (FPN) is significantly downregulated in malignant breast tissue, primarily due to elevated levels of hepcidin, the key regulator of iron homeostasis^[10]^. Hepcidin-induced degradation of ferroportin traps iron inside cancer cells, exacerbating intracellular iron overload and fueling tumor progression. Low ferroportin expression has been linked to increased metastatic potential and worse clinical outcomes in breast cancer patients^[7,8]^, Beyond its role in cellular metabolism, iron dysregulation actively contributes to the remodeling of the breast cancer tumor microenvironment. Tumor-associated macrophages (TAMs) accumulate iron and release pro-inflammatory cytokines, fostering an immunosuppressive environment that enables tumor cells to evade immune surveillance. Additionally, iron-rich conditions promote angiogenesis by enhancing the expression of vascular endothelial growth factor (VEGF), thereby supporting the formation of new blood vessels essential for tumor expansion and metastasis^[8]^. Oxidative stress, a hallmark of cancer, is exacerbated by excess iron in breast cancer cells. Through the Fenton reaction, iron catalyzes the production of reactive oxygen species (ROS), leading to oxidative DNA damage, genomic instability, and the activation of oncogenic pathways. High ROS levels further facilitate epithelial-mesenchymal transition (EMT), a key process in cancer metastasis that enhances the invasive and migratory capabilities of breast cancer cells. These oxidative stress-driven mechanisms contribute to tumor heterogeneity and the development of aggressive breast cancer phenotypes^[11]^.

Emerging evidence also suggests a complex interplay between iron metabolism and therapy resistance in breast cancer^[12]^. Elevated iron levels in tumor cells have been linked to resistance against chemotherapy and targeted therapies, partly due to enhanced DNA repair mechanisms and increased cellular antioxidant capacity. Furthermore, cancer cells exploit iron-related pathways to evade ferroptosis, a form of iron-dependent programmed cell death. This resistance to ferroptosis represents a major challenge in the development of iron-targeted therapies for breast cancer treatment^[13]^. Given the profound impact of iron metabolism on breast cancer biology, novel therapeutic strategies aimed at modulating iron homeostasis are being actively investigated. Iron chelators, such as deferoxamine (DFO) and deferasirox (DFX), have shown promise in limiting iron availability to tumor cells and suppressing their proliferation. Additionally, ferroptosis-inducing agents, including erastin and RSL3, are being explored as potential therapeutic options to selectively target iron-overloaded breast cancer cells. However, further research is needed to optimize these treatments while minimizing off-target effects and systemic toxicity^[13]^.

## Aim

The aim of this review is to explore the role of iron metabolism in breast cancer, emphasizing its mechanisms and therapeutic implications.

## Justification of the review

Breast cancer remains a major global health challenge, accounting for a significant proportion of cancer-related morbidity and mortality among women. Despite advancements in early detection and treatment, many patients continue to experience disease recurrence and resistance to conventional therapies. Among these metabolic alterations, iron dysregulation has emerged as a critical factor influencing tumor growth, metastasis, and therapy resistance. However, the role of iron metabolism in breast cancer remains underexplored compared to other metabolic pathways such as glucose and lipid metabolism. This review aims to bridge this knowledge gap by providing a comprehensive analysis of iron homeostasis in breast cancer and its potential therapeutic implications^[1,2]^. Iron metabolism is uniquely altered in breast cancer cells, creating an environment that favors tumor survival and proliferation. Increased iron uptake, elevated ferritin levels, and reduced iron export contribute to intracellular iron accumulation, promoting oxidative stress, DNA damage, and immune evasion. These iron-driven processes play a pivotal role in shaping breast cancer aggressiveness and treatment outcomes, particularly in aggressive subtypes such as TNBC. Given these findings, targeting iron metabolism presents a promising avenue for breast cancer therapy. However, a clear understanding of how iron-targeting agents can be integrated into current treatment regimens is still lacking. This review aims to critically evaluate the molecular mechanisms linking iron dysregulation to breast cancer pathogenesis and explore the potential of iron-based therapeutic interventions^[3,4]^. Furthermore, while iron-targeting strategies such as chelators, ferroptosis inducers, and iron-modulating nanoparticles have shown promise in preclinical studies, their clinical translation remains a challenge. Issues such as tumor selectivity, systemic toxicity, and resistance mechanisms need to be addressed to maximize the therapeutic benefits of iron modulation in breast cancer. By synthesizing existing knowledge on iron metabolism and highlighting emerging therapeutic strategies, this review seeks to provide a solid foundation for future research and clinical applications. A deeper understanding of iron regulation in breast cancer could pave the way for novel, more effective treatment approaches, ultimately improving patient outcomes and survival rates^[5,6]^.

## Review methods

### Literature search strategy

A comprehensive search was conducted across several academic databases, including PubMed, Scopus, and Google Scholar, to identify studies published between 2010 and 2024. The search terms included “iron metabolism,” “breast cancer,” “iron dysregulation,” “therapeutic implications,” “iron chelation,” and “ferroptosis.” The inclusion criteria were studies that explored the molecular mechanisms of iron metabolism in breast cancer cells, the impact of iron dysregulation on tumor progression, and experimental or clinical trials investigating iron-targeted therapies. Only studies published in peer-reviewed journals were included.

### Study selection and inclusion criteria

Articles were selected based on their relevance to the role of iron in breast cancer. Studies focusing on iron homeostasis in cancer, the molecular mechanisms involving iron uptake, storage, and export, and the potential of therapeutic interventions targeting iron were prioritized. Both preclinical and clinical studies were considered, provided they met the following inclusion criteria:

- Exploration of iron’s role in breast cancer biology.
- Studies on therapeutic approaches targeting iron metabolism in cancer treatment.
- Articles published in English. Studies were excluded if they were not directly related to breast cancer, lacked robust experimental or clinical evidence, or focused on non-cancerous conditions.

### Mechanisms of iron metabolism in breast cancer

Iron is an essential micronutrient that plays a fundamental role in cellular processes, including DNA synthesis, oxygen transport, and energy production. However, in breast cancer, iron metabolism is significantly altered, creating an environment that supports tumor growth, metastasis, and therapy resistance. Unlike normal cells, which tightly regulate iron homeostasis, breast cancer cells exploit iron regulatory pathways to increase iron availability, fueling their aggressive proliferation and survival^[14,15]^.

### Increased iron uptake in breast cancer

Breast cancer cells enhance iron acquisition primarily through the overexpression of TfR1, a key protein responsible for transferrin-bound iron uptake. TfR1 binds to transferrin and facilitates the internalization of iron through receptor-mediated endocytosis. Studies have shown that TfR1 expression is significantly upregulated in aggressive subtypes such as TNBC, leading to increased iron import. This iron accumulation supports DNA replication and cell cycle progression, allowing tumors to grow uncontrollably. Additionally, lactoferrin receptors, which facilitate alternative iron uptake, are also upregulated in some breast cancer cells, further increasing iron influx^[12,16,17]^.

### Iron storage and sequestration

Once inside the cell, iron is either utilized in metabolic pathways or stored within ferritin, an iron-binding protein that prevents toxic iron buildup. Breast cancer cells exhibit elevated ferritin levels, which serve as an adaptive mechanism to store excess iron while protecting against iron-induced oxidative damage. Increased ferritin expression has been correlated with poor prognosis, as it allows cancer cells to maintain iron reserves that support long-term survival and resistance to oxidative stress. Notably, ferritin not only serves as an intracellular iron buffer but also contributes to chemotherapy resistance by neutralizing the effects of iron-mediated cell death^[10,18]^.

### Reduced iron export in tumor cells

Iron export is primarily regulated by ferroportin (FPN), the only known iron-exporting protein. In breast cancer, FPN expression is often downregulated, leading to iron retention within tumor cells. The loss of FPN is largely mediated by increased levels of hepcidin, a liver-derived hormone that binds to FPN and triggers its degradation. Elevated hepcidin expression in breast cancer contributes to intracellular iron accumulation, further promoting tumor growth. Clinically, patients with low FPN and high hepcidin levels have been associated with worse survival outcomes, emphasizing the importance of iron retention in breast cancer progression^[19,20]^.

### Iron and oxidative stress in breast cancer

Excess iron in breast cancer cells undergoes redox cycling, generating high levels of reactive oxygen species (ROS) through the Fenton reaction. ROS play a dual role in tumor biology: at moderate levels, they promote DNA damage, genomic instability, and cell signaling pathways that drive proliferation and metastasis. Key pathways activated by ROS include NF-κB, MAPK, and PI3K/AKT, all of which contribute to tumor survival. However, excessive oxidative stress can trigger cell death, and breast cancer cells counteract this by upregulating antioxidant defenses such as glutathione peroxidase 4 (GPX4) and ferritin. This delicate balance between oxidative stress and antioxidant response enables cancer cells to thrive in iron-rich conditions^[21–23]^.

### Iron and the tumor microenvironment

Iron metabolism in breast cancer extends beyond individual tumor cells to influence the tumor microenvironment (TME). TAMs act as an iron reservoir, releasing iron into the extracellular space, which is then taken up by cancer cells. Iron-loaded macrophages also secrete pro-inflammatory cytokines, fostering an immunosuppressive microenvironment that enhances tumor growth and immune evasion. Additionally, iron supports angiogenesis, the formation of new blood vessels, by stabilizing hypoxia-inducible factor 1-alpha (HIF-1α), which promotes the expression of VEGF. This facilitates increased nutrient and oxygen supply to the tumor, sustaining its rapid expansion^[24,25]^.

### Iron and therapy resistance in breast cancer

Iron metabolism also plays a crucial role in breast cancer therapy resistance. High iron levels contribute to resistance against chemotherapy and radiation by enhancing DNA repair mechanisms. For instance, cisplatin-resistant breast cancer cells have been shown to upregulate iron storage proteins to mitigate oxidative stress. Furthermore, breast cancer cells develop resistance to ferroptosis, an iron-dependent form of programmed cell death, by increasing the expression of GPX4 and other antioxidants. This ability to evade ferroptosis represents a major challenge in iron-targeted therapy development^[26]^.

### Iron and hormone signaling in breast cancer

In estrogen receptor-positive (ER+) breast cancer, estrogen influences iron metabolism by increasing TfR1 expression, leading to greater iron uptake and storage. This suggests that iron metabolism is closely linked to hormone-driven tumor progression. Studies have indicated that estrogen-mediated iron retention supports the growth of ER+ breast cancer cells, highlighting the need to consider iron-targeting strategies alongside hormonal therapies^[27]^.

### Targeting iron metabolism as a therapeutic strategy

**Figure 1.**
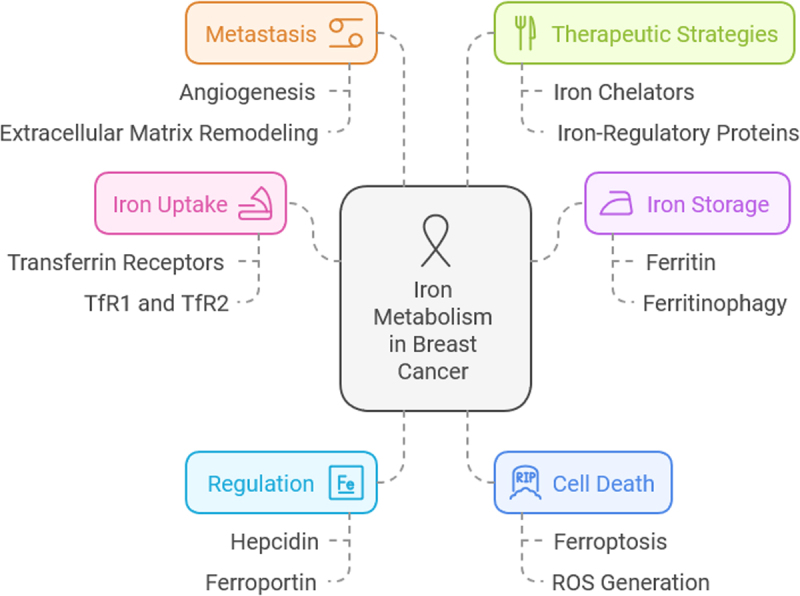
Mechanisms of iron metabolism in breast cancer. Source: Author.

Given the profound impact of iron dysregulation in breast cancer, researchers are actively exploring iron-modulating therapies. Iron chelators such as deferoxamine (DFO) and deferasirox (DFX) have been investigated for their ability to reduce intracellular iron levels and inhibit tumor growth. Additionally, ferroptosis-inducing agents such as erastin and RSL3 have shown promise in selectively targeting iron-overloaded breast cancer cells. However, challenges remain in optimizing these treatments while minimizing off-target effects^[28,29]^ (Fig. 1).

### Therapeutic implications of targeting iron metabolism in breast cancer

Targeting iron metabolism in breast cancer presents a promising avenue for therapeutic intervention due to the crucial role iron plays in tumor growth, metastasis, and therapy resistance. Given that breast cancer cells exhibit increased iron uptake, reduced iron export, and altered iron storage, disrupting these pathways can effectively inhibit tumor progression. Current research focuses on iron chelators, ferroptosis inducers, and iron-targeting drugs to exploit cancer cells’ iron dependency while minimizing toxicity to normal cells^[30,31]^.

### Iron chelation therapy

Iron chelators such as deferoxamine (DFO), deferasirox (DFX), and deferiprone (DFP) have been extensively studied for their ability to limit intracellular iron availability, thereby inhibiting tumor cell proliferation. These agents work by binding to free iron and reducing its participation in oxidative stress and DNA synthesis. In breast cancer models, DFO has been shown to suppress cell cycle progression and induce apoptosis, particularly in TNBC, which is highly iron-dependent. However, systemic iron depletion poses risks such as anemia and immune suppression, necessitating strategies to selectively target tumor-associated iron without disrupting normal iron homeostasis^[32,33]^.

### Targeting TfR1 for iron uptake inhibition

Breast cancer cells overexpress TfR1 to increase iron uptake, making TfR1 an attractive therapeutic target. Monoclonal antibodies such as ferristatin II and TfR1-blocking peptides are being investigated to disrupt iron acquisition. Additionally, TfR1-targeted drug conjugates, which combine cytotoxic agents with transferrin ligands, have demonstrated potential in selectively delivering chemotherapy to iron-dependent cancer cells. Clinical trials are ongoing to evaluate the efficacy of TfR1-targeted therapies in aggressive breast cancer subtypes^[16]^.

### Ferroptosis induction as an anti-cancer strategy

Ferroptosis, an iron-dependent form of programmed cell death, is an emerging therapeutic approach in breast cancer. Unlike apoptosis, ferroptosis is characterized by lipid peroxidation and oxidative stress, which breast cancer cells counteract through high expression of GPX4 and ferritin. Small molecules such as erastin and RSL3 have been shown to trigger ferroptosis by inhibiting GPX4, leading to lethal iron-induced oxidative damage in breast cancer cells. Ferroptosis inducers are particularly effective in TNBC and drug-resistant breast cancer, where conventional therapies fail. However, the challenge lies in optimizing ferroptosis activation while preventing toxicity in normal tissues^[34]^.

### Hepcidin-ferroportin axis modulation

Hepcidin, a liver-derived peptide, regulates iron export by binding to ferroportin (FPN) and promoting its degradation. In breast cancer, hepcidin overexpression leads to intracellular iron retention, fostering tumor progression. Strategies to reduce hepcidin levels or restore FPN expression have shown potential in reversing iron overload in breast cancer cells. Small molecules that inhibit hepcidin expression or stabilize FPN are currently under investigation for their tumor-suppressive effects^[35]^.

### Combining iron-targeting therapies with conventional treatments

Iron metabolism-targeted therapies are increasingly being explored in combination with chemotherapy, radiation, and immunotherapy to enhance treatment efficacy. Studies suggest that iron chelation sensitizes breast cancer cells to doxorubicin and cisplatin, reducing drug resistance. Additionally, ferroptosis inducers have been shown to enhance the effects of radiotherapy by increasing oxidative stress, making tumors more susceptible to radiation-induced damage. Given the interplay between iron metabolism and the tumor microenvironment, integrating iron-targeted therapies with immune checkpoint inhibitors may also improve immune responses against breast cancer^[36]^.

### Nanotechnology-based iron therapy approaches

Advancements in nanomedicine have enabled the development of iron-based nanoparticles for targeted drug delivery. Iron oxide nanoparticles (IONPs) have demonstrated dual functionality in breast cancer therapy by acting as therapeutic agents and imaging tools. These nanoparticles can be engineered to release iron chelators or ferroptosis inducers directly into tumor cells, reducing systemic toxicity. Furthermore, magnetic iron nanoparticles are being explored for hyperthermia therapy, where localized heating induced by magnetic fields selectively destroys cancer cells while sparing healthy tissue^[37]^.

### Targeting iron-associated inflammation and the tumor microenvironment

**Figure 2.**
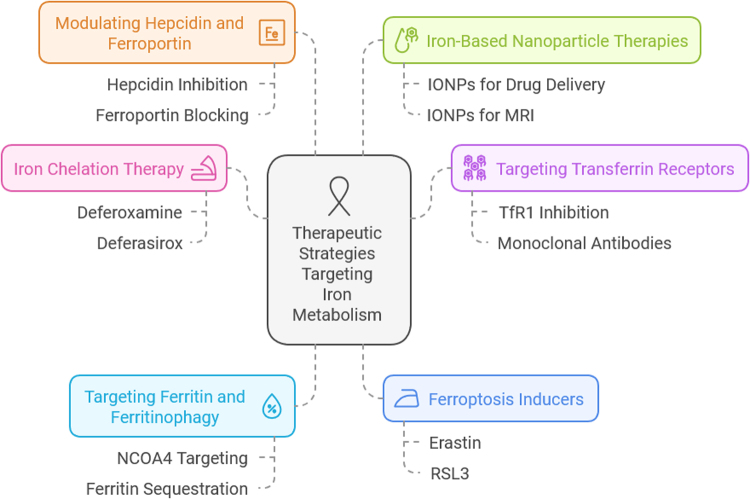
Therapeutic implications of targeting iron metabolism in breast cancer. Source: Author.

Iron metabolism is closely linked to TAMs, which create an immunosuppressive microenvironment by releasing excess iron. Strategies to reprogram macrophages toward an anti-tumor phenotype include the use of iron chelators, ferroportin stabilizers, and anti-inflammatory agents. Additionally, targeting hypoxia-inducible factor 1-alpha, which is stabilized by iron and promotes angiogenesis, may further limit tumor growth and metastasis^[32]^ (Fig. 2).

## Recommendations

1. Development of tumor-specific iron-targeting therapies: Future research should focus on designing breast cancer-specific iron chelators and transferrin receptor inhibitors to selectively target iron metabolism in tumor cells while sparing normal tissues. This will help minimize systemic toxicity and side effects such as anemia and immune suppression.
2. Integration of iron-targeted strategies with standard treatments: Clinical trials should explore the combination of iron-targeting agents with chemotherapy, radiotherapy, and immunotherapy to enhance treatment efficacy, especially in TNBC and drug-resistant breast cancer subtypes. Iron chelation may improve drug sensitivity, while ferroptosis induction could increase radiation effectiveness.
3. Identification of biomarkers for iron metabolism dysregulation: The discovery of reliable biomarkers of iron overload and iron-dependent tumorigenesis in breast cancer could facilitate early diagnosis, prognosis prediction, and personalized treatment approaches. Monitoring iron-related proteins such as ferritin, TfR1, ferroportin, and hepcidin may help tailor therapeutic strategies.
4. Advancement of ferroptosis-based therapies: Ferroptosis induction represents a promising therapeutic avenue for iron-dependent breast cancer cells, particularly in aggressive and treatment-resistant cases. More research is needed to optimize ferroptosis inducers and identify combination strategies that enhance ferroptotic cell death without affecting normal tissues.
5. Exploration of nanotechnology for targeted iron modulation: The use of IONPs and other nanocarriers should be further explored for targeted drug delivery, imaging, and hyperthermia therapy in breast cancer. These approaches may help overcome the limitations of traditional iron-targeting drugs by improving specificity and reducing systemic side effects.
6. Modulation of the hepcidin-ferroportin axis: Given the role of hepcidin overexpression in breast cancer progression, research should focus on developing hepcidin inhibitors or ferroportin stabilizers to restore iron homeostasis and suppress tumor growth. Such interventions could complement existing breast cancer treatments.
7. Targeting iron-associated tumor microenvironment factors: Future therapies should address iron-driven inflammation and immune suppression in the breast cancer microenvironment. Strategies to reprogram TAMs and modulate iron availability could enhance anti-tumor immunity and improve treatment outcomes.
8. Personalized approaches based on iron metabolism profiles: Since breast cancer patients exhibit varying levels of iron metabolism dysregulation, individualized treatment plans based on genetic, epigenetic, and metabolic profiling should be developed. Personalized iron-targeting therapies could improve efficacy while minimizing adverse effects.
9. Clinical trials for iron-targeting agents: There is a need for well-designed clinical trials to assess the safety, efficacy, and long-term benefits of iron-modulating therapies in breast cancer. Trials should evaluate both monotherapies and combination treatments to determine the most effective strategies for different breast cancer subtypes.
10. Public health awareness and nutritional interventions: Since iron metabolism is influenced by diet and systemic iron levels, awareness campaigns should educate breast cancer patients on safe dietary iron intake. Nutritional interventions tailored to individual iron metabolism status may help optimize patient outcomes and complement medical treatments.

## Conclusion

Iron metabolism plays a pivotal role in breast cancer progression, influencing tumor growth, metastasis, and resistance to therapy. Breast cancer cells exploit iron for increased proliferation by enhancing iron uptake through transferrin receptor overexpression, suppressing iron export via ferroportin downregulation, and utilizing iron storage proteins such as ferritin to prevent oxidative damage. These adaptations create an iron-rich microenvironment that fuels tumorigenesis and therapy resistance, making iron homeostasis a critical target for novel therapeutic interventions. Emerging treatment strategies, including iron chelation therapy, ferroptosis induction, and transferrin receptor-targeted therapies, have demonstrated promising anti-tumor effects in preclinical models. Additionally, modulation of the hepcidin-ferroportin axis and nanotechnology-based iron-targeting approaches offer innovative avenues to selectively disrupt iron availability in breast cancer cells while minimizing systemic toxicity. The combination of iron-targeted therapies with chemotherapy, immunotherapy, and radiation may enhance treatment efficacy, particularly in aggressive subtypes such as triple-negative breast cancer (TNBC).

## Data Availability

Not applicable as this is a review.

## Research registration unique identifying number (UIN)

Not applicable as this is a review.
